# Functional haematopoietic progenitor cells in the adult human lung

**DOI:** 10.21203/rs.3.rs-3576483/v1

**Published:** 2023-11-28

**Authors:** Mark Looney, Catharina Conrad, Melia Magnen, Jessica Tsui, Harrison Wismer, Mohammad Naser, Urmila Venkataramani, Bushra Samad, Simon Cleary, Longhui Qiu, Jennifer Tian, Marco De Giovanni, Nicole Mende, Emmanuelle Passegue, Elisa Laurenti, Alexis Combes

**Affiliations:** University of California, San Francisco; University of California, San Francisco; University of California, San Francisco; University of California, San Francisco; University of California, San Francisco; UCSF; University of California, San Francisco; University of California, San Francisco; UCSF; University of California, San Francisco; University of California, San Francisco; University of California, San Francisco; Wellcome MRC Cambridge Stem Cell Institute, University of Cambridge, Cambridge, UK; Columbia University Irving Medical Center; University of Cambridge; University of California, San Francisco

## Abstract

The bone marrow is classically viewed as the main site of blood cell production in adults, however, rare pools of haematopoietic stem and progenitor cells with self-renewal and differentiation potential have been described in extramedullary organs. The lung is primarily known for its role in gas exchange but has recently been identified as a site of blood (platelet) production in mice. Here, we show that functional haematopoietic precursors reside in the extravascular spaces of the human lung, at a frequency similar to the bone marrow, and are capable of proliferation and engraftment. Comparative transcriptomic profiling of pulmonary and medullary CD34^+^ haematopoietic progenitor cells revealed organ-specific gene signatures and indicated greater baseline activation of immune, megakaryocyte/platelet and erythroid-related pathways in progenitors from the lung. In the lung, these cells predominately reside in the vascular-rich alveolar interstitium and near macrophages. These results identify the lung as an important niche for uniquely programmed blood stem and progenitor cells with considerable potential to support haematopoiesis in humans.

## Introduction

Haematopoietic stem cells (HSCs) are self-renewing cells residing in the bone marrow (BM) that are ultimately responsible for production of all mature circulating blood cell lineages^1^. Despite the burden of maintaining haematopoiesis, HSCs are a rare cell type in the BM accounting for <0.01% of nucleated cells^2,3^. They occupy and are maintained by a specific BM niche yet are able to exit the BM environment and enter the circulation, although the explanation of this behavior is largely unknown^4^. HSCs are central to the pathogenesis of serious disorders including myelodysplasia, acute and chronic leukemia, aplastic anemia^5^ and clonal hematopoiesis^6^, and HSC transplantation can be a life-saving therapy. Previously, we discovered extravascular haematopoietic stem and progenitor cells (HSPCs) in the adult mouse lung capable of engraftment in the BM and multi-lineage haematopoiesis^7^. Motivated by this background, in this study we sought to determine if HSPCs occupied the adult human lung—an organ with a vast vasculature that contains a wide-ranging repertoire of stromal and immune cells.

## The adult human lung contains functional HSPCs

**Figure 1:**
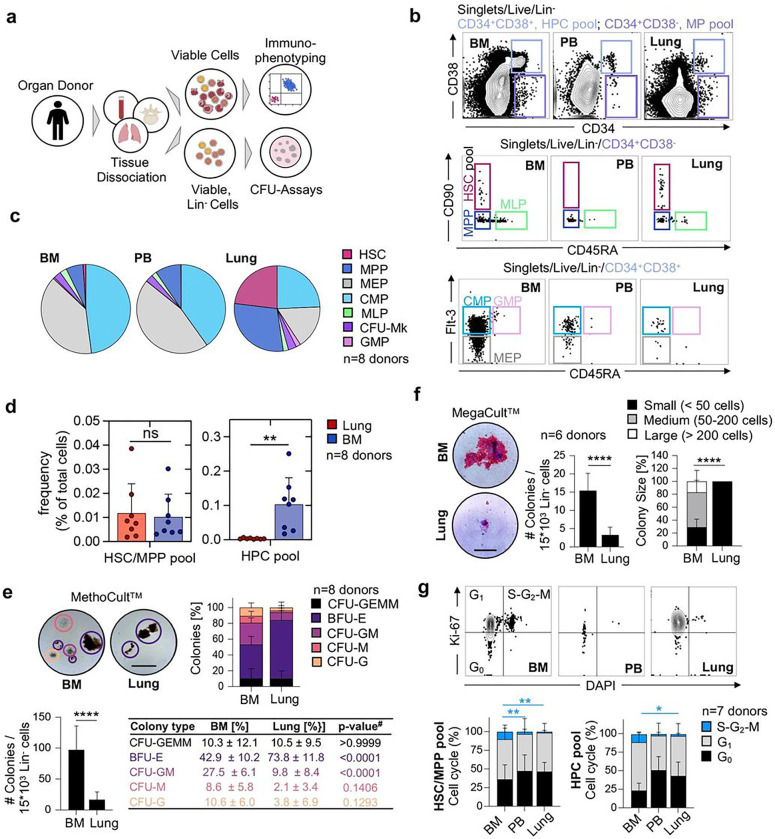
The human lung contains phenotypic haematopoietic progenitors with *in vitro* proliferation and differentiation capacity. **(a)** Pipeline for flow-cytometric immunophenotyping and evaluation of *in vitro* colony-forming capacity of haematopoietic progenitor cells from BM, lung and PB of organ donors. **(b)** Normalized flow cytometry plots of BM, PB in the Live/Lin^−^ gate from a representative donor showing stem cell subsets within the multipotent (MP [CD34^+^CD38^−^], light purple) and the haematopoietic progenitor cell (HPC [CD34^+^CD38^+^], light blue) pool. HSC, haematopoietic stem cell; MPP, multipotent progenitor; MLP, multilymphoid progenitor; CMP, common myeloid progenitor; MEP, megakaryocyte-erythroid progenitor; GMP, granulocyte-macrophage progenitor; CFU-Mk, colony-forming-unit megakaryocyte. **(c)** Composition of haematopoietic progenitor subsets in the BM, PB and lung (n=8). **(d)** Frequency of cells in the HSC/MPP and HPC pool as a percentage of total nucleated cells in the lung or BM, respectively. Individual values are shown, bars represent mean ± SD. Student’s t-test, **p<0.01; ns, not significant. **(e)** Culture initiating capacity of lung and BM progenitors in MethoCult^™^ (n=8): Representative colonies (scale bar, 500μm), colony composition and colony quantity for progenitors derived from the BM and lung. Student’s t-test, ****p<0.0001, ^#^ANOVA followed by Sidak’s multiple comparison test. CFU, colony-forming unit; BFU-E (purple), burst-forming unit-erythroid; G (orange), granulocyte; M (red), macrophage; GM (pink), granulocyte macrophage; GEMM (black), granulocyte, erythroid, macrophage, megakaryocyte. **(f)** Culture initiating capacity of lung and BM progenitors in MegaCult^™^ (n=6): Representative colonies (scale bar, 100μm), colony quantity and colony size for progenitors from the BM and lung. Bar graph represents mean number of colonies ± SD, Student’s t-test, ****p<0.0001. Stacked bars represent mean proportion ± SD, Kruskal-Wallis test, ****p<0.0001. **(g)** Proportions of cycling (S-G2-M phase (blue), Ki-67^+^DAPI^+^), preparing/growing (G1 (grey), Ki-67+DAPI^−^) and resting cells (G0 (black), Ki-67^−^DAPI^−^) in the HSC/MPP and HPC pool from BM, PB and lung (n=7). Stacked bars represent mean proportion ± SD, ANOVA followed by Sidak’s multiple comparison test, **p<0.01, *p<0.05. For comparisons not indicated, no statistically significant differences were observed.

We had the unique opportunity to receive matched adult human lungs, vertebral bodies (BM), and peripheral blood (PB) from deceased research donors (Extended Data Table 1). The lungs were extensively perfused at the time of collection, and we selected healthy-appearing lung tissue from the upper lobes for our experiments (Extended Data Fig. 1a). After tissue dissociation and the rendering of single-cell suspensions, we first characterized live, lineage negative (Lin^−^) cells (Fig. 1a)using standard surface markers for HSPCs (Extended Data Table 2)^8–10^. Notably, our lineage panel contained markers for mature immune, endothelial, and epithelial cells, allowing for the phenotyping of rare Lin^−^CD34^+^ cells. We discovered a distinct population of multipotent progenitors (MP; (CD34^+^/CD38^−^) in the lung and BM and very few of these cells in the peripheral blood (PB) (Fig. 1b). The lung and BM contained cells with surface staining consistent with HSCs (CD34^+^/CD38^−^/CD90^+^/CD45A^−^) but these cells were absent in the PB. Multipotent progenitor (MPP) cells (CD34^+^/CD38^−^/CD90^−^/CD45A^−^) were observed in all three tissues. CMP (CD34^+^/CD38^+^/CD45A^−^/Flt-3^+^), GMP (CD34^+^/CD38^+^/CD45A^+^/Flt-3^+^), and MEP (CD34^+^/CD38^+^/CD45A^−^/Flt-3^−^) cells were observed in all three tissues but were much less common in the lung (Fig. 1b). Overall, the BM and PB had nearly identical proportions of haematopoietic progenitors, while Lin^™^ lung cells were enriched for immunophenotypic HSCs and MPPs (Fig. 1c). To rule out residual blood as a source of HSPCs in perfused lungs, we estimated the numbers of progenitors in equivalent volumes of blood and lung tissue, calculating lung HSPC numbers that could not be explained by retention of intravascular blood in lungs (Extended Data Fig. 1b). Further, the distinct proportions of cell subsets in lung and blood indicates a tissue-specific progenitor composition (Extended Data Fig. 1c). Remarkably, the frequency of the immunophenotypic HSC/MPP pool in the lung is similar to the BM (Fig. 1d), while the pool of more committed haematopoietic progenitor cells is much smaller in the lung (Fig. 1d, Extended Data Fig. 1d). We also tested for the effects of donor age and gender on our results and found that increasing age was associated with fewer numbers of HSPC pools in the BM but not in the lung (Extended Data Fig. 2a-b). Gender was not associated with changes in HSPC frequency in the BM or lung (Extended Data Fig. 2c-d).

Since fibroblasts are generally lineage-negative cells, but some can be CD34^+ 11^, we included a marker for fibroblasts (PDGFRα) in our lineage panel to determine if fibroblast contamination could affect our results, but we did not detect any changes to HSPC frequencies between our two lineage panels (Extended Data Fig. 3a-c).

We next tested the functional capacity of lung HSPCs using *in vitro* colony forming assays. We plated Lin^−^ cells from the lung or BM in MethoCult^™^ and observed a variety of colonies from both tissues. The Lin^−^ cells from the lung produced overall fewer colonies, but with a significant increase in the relative proportion of erythroid colonies (BFU-E) (Fig. 1e). Following morphologic colony assessment, we used flow cytometry (Extended Data Fig. 4a-b) to confirm cellular colony composition and found that lung colonies were enriched with cells expressing the erythrocyte marker GlyA (Extended Data Fig. 4c). We also plated Lin^−^ cells in MegaCult^™^ to test the potential to produce megakaryocytes, a cell population that we previously described as resident cells in the mouse lung^7^. Both BM and lung cells were capable of producing megakaryocyte colonies although the lung produced fewer and small colonies (Fig. 1f). The fewer colonies observed from lung cells likely relates to overall reduced cell cycling compared to the BM (Fig. 1g). Together, we conclude that the human lung is enriched in functional HSPCs exhibiting an erythroid bias.

## Human lung-derived haematopoietic progenitors have engraftment potential

**Figure 2:**
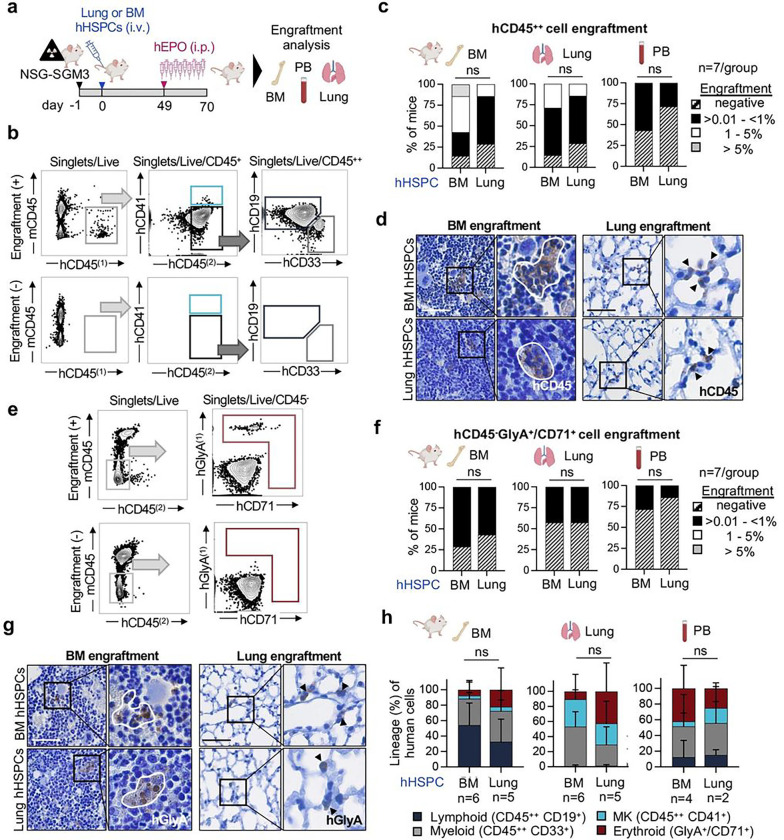
Human lung-derived haematopoietic progenitors have in vivo engraftment potential. **(a)** Experimental protocol to compare the *in vivo* engraftment efficiency of lung and BM haematopoietic progenitors: After sublethal irradiation, NSG-SGM3 mice were injected i.v. with either 1.5 ×10^6^ Live/Lin^−^ human lung or BM cells. 10 weeks post transplantation, the BM, PB and lung of recipient mice were collected and investigated for human cell engraftment. **(b)** Representative flow plots of human myeloid (hCD45^++^, hCD33^+^) and lymphoid (hCD45^++^, hCD19^+^) cell engraftment in the BM of a recipient mouse (+, upper panel) and non-transplanted control (−, lower panel). **(c)** Engraftment efficiency of human cells in the BM, lung and PB of recipient mice after xenotransplantation of HSPCs from either the human BM or lung (n=7/group, % relative to CD45^+^ cells). Fisher’s exact test, ns, not significant. **(d)** Detection of human cells in the BM (left panel) and lung (right panel) of recipient mice by immunostaining against human CD45 (hCD45). Scale bar, 50μm. **(e)** Representative flow cytometry plots of human erythroid (CD45^−^, hGlyA^+^ or hCD71^+^) cell engraftment in the BM of a recipient mouse (+, upper panel) and non-transplanted control (−, lower panel). **(f)** Human erythroid cell expansion (CD45^−^, hGlyA^+^ or hCD71^+^) in the BM, lung and PB of recipient mice after xenotransplantation with HSPCs from either the human BM or lung (n= 7/group, % relative to CD45^−^ cells). Fisher’s exact test, ns, not significant. **(g)** Detection of human erythroid cells in the BM (left panel) and lung (right panel) of recipient mice by immunostaining against human GlyA (hGlyA). Scale bar, 50μm. **(h)** Proportion of lineage expansion across all human cells detected in the BM, lung and PB, respectively. Stacked bars represent mean proportion ± SD, ANOVA followed by Sidak’s multiple comparison test, ns, not significant.

We next tested whether HSPCs isolated from the lung are capable of engraftment when xenotransplanted into immunodeficient mice^12^. We chose NSG-SGM3 mice (human SCF, GM-CSF, IL-3)^12^ for these experiments in which we adoptively transferred magnetic-enriched Lin^−^ cells from the lung or BM into mice with sublethal irradiation and assessed engraftment at day 70. Mice also received recombinant human erythropoietin injections in the final 3 weeks of the experiment (Fig. 2a). We rigorously defined engraftment as the presence of human CD45^+^ cells positive for two different antibody clones with the threshold for engraftment set to ≥ 0.01 % CD45^++^ cells of all CD45^+^ (mouse and human) with at least 30 cells recorded in the CD45^++^ gate for BM, lung and spleen, and ≥ 15 cells for PB^12^. Examples of positive and negative engraftment (sublethal irradiation without adoptive cell transfer) are shown in Fig. 2b. Overall, 6/7 mice with BM-derived HSPCs and 5/7 mice with lung-derived HSPCs engrafted in the BM and in the lung tissue (Fig. 2c, Extended Data Fig. 5a-b). In the PB, engraftment was observed in 4/7 mice with BM-derived HSPCs and 2/7 mice with lung-derived HSPCs (Fig. 2c). Examples of BM- or lung-derived cells in the mouse BM or lung are shown in Fig. 2d. Given our observations of a potential erythroid bias in lung HSPCs, we next determined erythroid engraftment using the gating strategy in Fig. 2e to detect CD45^−^, hGlyA^+^, hCD71^+^ cells. We observed similar erythroid engraftment of BM- or lung-derived HSPCs in the mouse BM, lung, or PB (Fig. 2f–g). Similar to BM-derived HSPCs, lung-derived HSPCs were capable of multilineage blood cell production (Fig. 2h, Extended Data Fig. 5c-e).

## Human lung HSPCs have unique transcriptional programming

**Figure 3:**
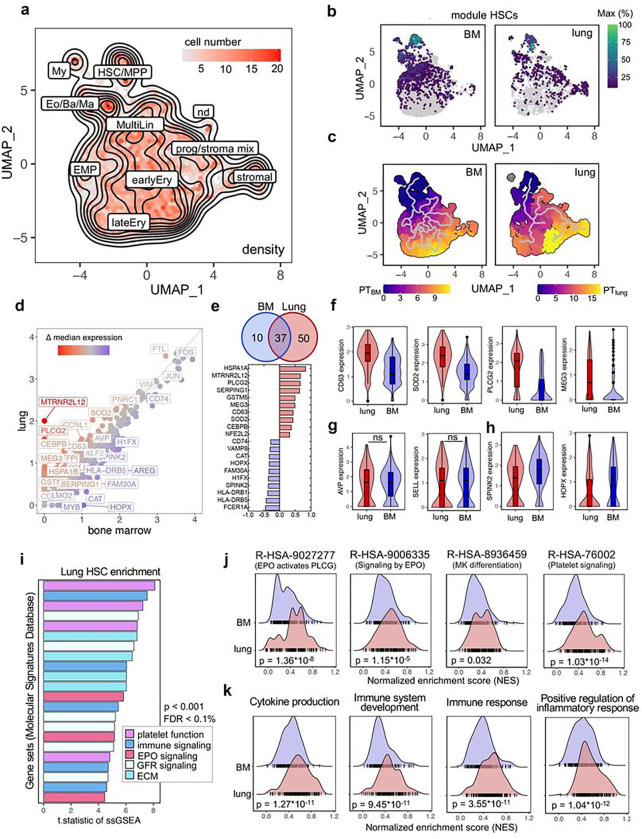
Comparative transcriptomic analysis of lung and BM HSCs reveals shared and unique gene expression profiles. (a) Annotated, batch-corrected UMAP projection with cell density representation of merged BM and lung Lin^−^CD34^+^ progenitor subsets from 8 human donors. HSC/MPP, haematopoietic stem cell/multipotent progenitor; My, myeloid cell; Eo/Ba/Ma, eosinophil/basophil/mast cell progenitor; MultiLin, multi-lineage; EMP, erythroid megakaryocytic progenitor; earlyEry, early erythroid progenitor; lateEry, late erythroid progenitor; prog/stroma mix, progenitor stroma cell mix; nd, not determined. **(b)** Grouping of gene expression patterns into modules using Monocle3. Aggregate expression values of genes in the module highly specific for HSCs (Extended Data Figure 6) are shown individually for the BM and lung. **(c)** Pseudotime calculation for each cell within the BM and lung using Monocle3 to infer progression through different cellular differentiation to provide insights into the developmental trajectory. **(d)** Scatter plot of median gene expression of cells in the HSC/MPP cluster from the lung (red) and BM (blue) to visualize consistent (grey) and differentially (highlighted) expressed genes. **(e)** Venn diagram and top 10 differentially expressed genes. The number in each circle represents the amount of differentially expressed genes between lung (red) and BM (blue), the overlapping number indicates mutual differentially expressed genes based on the Wilcoxon ran-sum test in Seurat’s ‘FindMarkers’ function. **(f)** Box and violin plots showing the distribution of selected genes upregulated in pulmonary haematopoietic progenitor cells. Wilcoxon adjusted p-value <0.001. **(g)** Selection of marker genes shared between lung and BM as box and violin plots, respectively. ns, not significant. **(h)** Box and violin plots showing the distribution of markers genes upregulated in BM HSCs, Wilcoxon adjusted p-value <0.001. **(i)** T.statistic of ssGSEA scores for selected gene sets (Hallmark, Reactome, Biocarta, KEGG) enriched in pulmonary HSCs categorized by recurring functions. EPO, erythropoietin; ECM, extracellular matrix, FDR, false discovery rate; GFR, growth factor receptor; ssGSEA, single-sample Gene Set Enrichment Analysis. **(j)** Enrichment ridge plots comparing the distribution of enrichment scores in HSCs from lung (red) and BM (blue) of selected Reactome pathways. Rug plots indicate the scores of individual cells along the ridge plot. **(k)** Enrichment ridge plots showing the distribution of enrichment scores in lung (red) and BM (blue) with individual cell placement on the rug plot to compare selected GOBP (Gene Ontology Biological Process) gene set enrichments.

The availability of matched lung and medullary HSPCs allowed us to directly compare their transcriptional profiles using single-cell RNA-sequencing (scRNA-seq, Extended Data Fig. 6). We integrated all samples using Harmony^14^ and generated a batch corrected UMAP to find clusters of transcriptionally similar cells. By using the function ‘findConservedMarkers’ we identified genes that were consistently expressed across BM and lung-derived cells and annotated clusters based on a reference data set^15,16^. Dimensionality reduction yielded a visual representation consistent with HSC and MPP production of progenies with progressive commitment to more differentiated fates (Fig 3a, Extended Data Fig. 6a-b). To validate our annotation, we generated co-regulated modules of differentially expressed genes using Monocle3^17^. The module highly specific for HSCs contains genes such as *AVP, SPINK2, SELL* and *HOPX* and was strongly expressed in cells from both the BM and lung (Fig. 3b, Extended Data Fig 6c-d). We ordered the cells along a pseudotime trajectory to reconstruct their developmental path for each tissue individually and found a relationship between HSCs and stromal cells in the lung that was absent in cells derived from the BM (Fig. 3c). As previously discussed, some pulmonary fibroblasts are CD34^+^ and these results could point to the ontogeny of a subset of lung stromal cells.

Next, we compared the differential gene expression between medullary and lung cells within the HSC/MPP cluster and plotted the median expression of both cells on a scatter plot (Fig 3d). Using the Wilcoxon rank-sum test in Seurat’s ‘FindMarkers’ we identified 50 genes that were upregulated in lung HSCs and 10 genes that were higher in BM cells (Fig. 3e). Among the top upregulated genes in lung HSCs, several genes (*CEBPB, SOD2, PLCG2, HSPA1A*) were associated with maintaining hematopoietic stem cell quiescence and fitness^18–21^ (Fig 3e). We attributed the highest biological relevance to genes that were upregulated in most of the cells, as validated by analyzing the distribution of gene expression values (Fig. 3f–h). HSCs from the lung have unique features (Fig. 3f) and share characteristics of the haematopoietic lineage (Fig. 3g), while as expected, cells from the BM have high expression of classical stem cell genes (Fig. 3h)^15^.

Next, we performed single-sample Gene Set Enrichment Analysis (ssGSEA) to identify pathways that are differentially regulated between cells from the lung and the BM. The enrichment scores were calculated across all individual cells and tissues for gene set collections from the Molecular Signature Database (H: hallmark, CP: canonical pathways, C5: ontology). In the gene sets analyzed, repeatedly pathways associated with erythropoietin (EPO) signaling, platelet function, and immune responses were found to be upregulated in pulmonary HSCs (Fig. 3i). Side-by-side comparison of selected pathways indicates that HSCs from the lung are enriched for megakaryocyte (R-HSA-8936459) and EPO-induced erythroblast (R-HSA-9027277, R-HSA-9006335) differentiation, as indicated by higher normalized enrichment scores (NES) (Fig. 3j). Our finding of increased EPO signaling pathways in lung HSCs is consistent with our data in Fig. 1e on the erythroid-biasing of lung colonies. Our finding of platelet and megakaryocyte-skewing of lung HSCs is provocative in light of our previous work on platelet production in the lung and tissue-resident immune-like megakaryocytes^7,22^. Additionally, we found inflammatory signaling to be upregulated in pulmonary HSCs (Fig. 3k), suggesting that these cells could impart unique immunological functions of their progeny.

Given that HSCs have not been previously documented in published human lung cell atlases, we used UCell scoring to identify potential HSCs based on their gene profile. In an analysis of integrated data from 9 human lung scRNA-seq studies (Human Lung Cell Atlas V2, HLCA)^23^, we found CD34^+^ cells predominately in endothelial, lymphatic and fibroblast clusters (Extended Data Fig. 7a-b). We identified Lin^−^, CD34^+^ cells with an HSC signature in the HLCA using UCell scoring (Extended Data Fig. 7c-d) and projected these cells on the UMAP structure of haematopoietic progenitors from the lung and BM (Fig. 3a) in Extended Data Fig. 7e. We mapped 43 cells to the HSC/MPP cluster (Extended Data Fig. 7f) across all integrated datasets in the HLCA (Extended Data Fig. 7g). We conclude that lung HSCs do not form distinct clusters when using standard unsupervised clustering techniques given their rarity and the co-expression of CD34 in multiple lung cell types.

## HSPCs in the lung reside in the extravascular tissue

**Figure 4:**
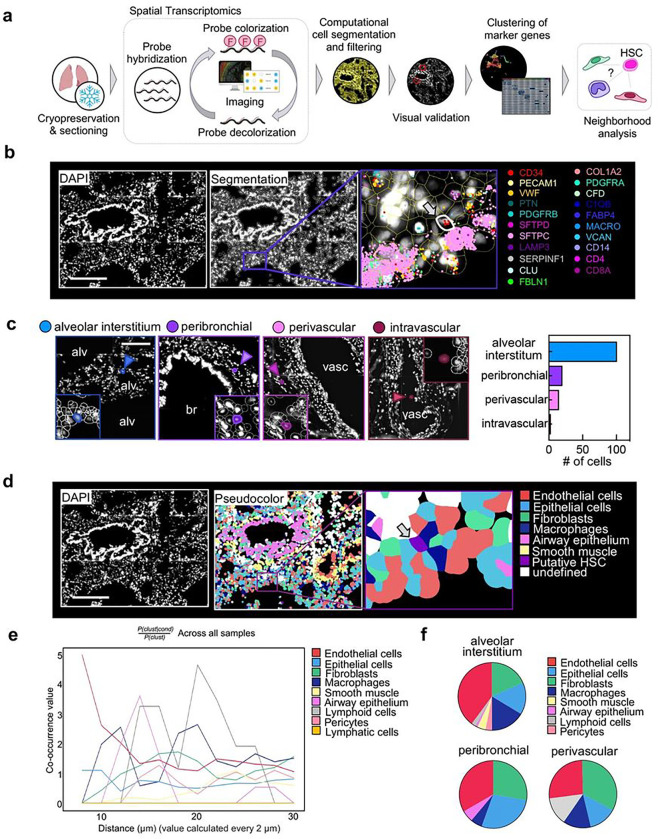
Spatial mapping of phenotypic CD34 + HSPCs in the lung. **(a)** Spatial transcriptomics analysis workflow. smFISH was performed to visualize gene expression in human lung tissue. Transcripts were assigned to individual cells after cell segmentation and putative HSPCs were computationally identified based on their gene signature and visually validated. Lung stromal and immune cells were annotated based on marker gene clusters (Extended Data Fig. 8). Subsequently, co-occurence analysis was performed. **(b)** Representative image of a putative HSPC in its pulmonary niche. Upper panel (left to right): DAPI staining, QuPath segmentation, zoom on putative HSC (arrow). Selected transcripts are shown. Scale bar, 250 μm **(c)** Anatomic location of candidate cells in the lung. Representative images of phenotypic HSPCs in four major locations (alveolar interstitium, peribronchial, perivascular or intravascular) and proportion of cells in each location. Alv, alveolar space; br, bronchus; vasc, vasculature. Scale bar, 150μm. **(d)** Pseudo-coloring of cell types in the lung tissue based on marker clustering (Extended Data Fig. 8). Zoom on putative HSPC in niche. Scale bar, 250 μm. **(e)** Squidpy co-occurrence score computed every 2 μm between putative HSPCs and the rest of the clusters across lung tissue sections from 4 organ donors. High score values indicate greater co-occurrence probability; endothelial cells (red) and macrophages (dark blue) co-occur with the HSPCs at short distances. **(f)** Pie graphs showing the proportion of neighboring cells within a radius of 20 μm from the putative HSPCs in the major anatomic locations.

The lung is composed of diverse cell entities, such as epithelial, endothelial, stromal, and immune cell subpopulations that could provide a unique niche for HSPC maintenance and differentiation^16^. To map HSPCs to their lung compartment, we used a spatial transcriptomics approach based on combinatorial single-molecule fluorescent *in situ* hybridization (Fig 4a). We designed a marker panel that characterizes HSPCs as well as the common cell entities of the lung (Extended Data Table 3). Following QuPath-based cell segmentation, we filtered for putative HSPCs defined by CD34^+^ positivity, expression of HSPC-associated transcripts and negativity for marker genes of other lung cell entities (Fig. 4b, Extended Data Fig. 8a, b). We visually validated all candidate cells and assigned them to their anatomic location. Over 90% of cells matching the criteria for HSPCs localized to the extravascular lung, with the majority of the cells in the alveolar interstitium or in proximity of bronchi (peribronchial) and vasculature (perivascular) (Fig. 4c). To define the neighborhood of HSPCs on the cellular level, we performed unsupervised clustering of the cells generated via segmentation using their transcript expression values (Fig. 4d, Extended Data Fig. 8c and Extended Data Fig. 9a-d). Based on this annotation, we used Squidpy for co-occurrence analysis across all samples^24^ suggesting that the immediate HSPC niche is mainly formed by endothelial cells and macrophages, although epithelium and fibroblasts have a steady presence (Fig. 4e–f).

## Discussion

For many years, HSCs were viewed as unbiased cells that initiated haematopoietic development and specialization. Enabled by new technologies, our understanding of haematopoiesis has been reframed to include the possibility that developmental biases may be present even in these most undifferentiated cells, such as with megakaryocyte-biased HSCs^9,25^. However, the mechanisms responsible for this early biasing or specialization are not clear^26,27^. Here, we propose that the traditional view of HSC residency in the BM should be reconsidered to include other tissues, such as the lung. Indeed, and remarkably, we found an equal frequency of HSPCs residing in the bone marrow and lung. This was surprising, especially given the absence of a distinct HSPC population despite extensive molecular profiling of the adult human lung^16^. We used approaches to enrich for the presence of HSPCs in our studies, which were not done in previous studies and likely enabled our detection of this rare subset of cells amongst the >30 different lung cell types.

It is clear from our studies that the lung HSPCs have unique features compared to their medullary counterparts, but also to HSPCs found at other extramedullary sites (peripheral blood and spleen^28^). Chief amongst these, and perhaps obvious from our understanding of lung biology, is that lung HSPCs, like spleen HSPCs, are less active in terms of cell division and the production of more differentiated and specialized hematopoietic cells than their BM counterparts. These results from *in vitro* experiments would suggest that perhaps lung HSPCs function as a reserve pool that could be mobilized in the setting of haematopoietic stress. Indeed, our xenotransplantation experiments indicated that lung HSPCs performed similarly to medullary HSPCs, at least in the short-term, when given the challenge of engraftment after sublethal irradiation. Our transcriptomics analysis revealed a distinct molecular program in lung HSPCs that was further distinguishing compared to medullary HSPCs. We found clear megakaryocyte biasing of lung vs. medullary HSPCs indicating that perhaps the lung is a source of these biased progenitors. This is an interesting finding given (1) the role of the lung in platelet biogenesis and (2) the presence of tissue resident immune-like megakaryocytes (of unclear ontogeny) in the lung^7^. We further found an erythroid bias of lung HSPCs, akin to that reported in peripheral blood and spleen HSPCs^28^. Erythroid bias therefore is a shared feature of steady-state extramedullary HSPCs, perhaps determined by distinct access to environmental oxygen versus the relatively hypoxic environment of the BM^29^.

Our spatial transcriptomic studies were essential in confirming the presence of HSPCs in the lung and defining their precise locations and niche. Given the prevailing dogma that HSCs widely circulate^30–32^, it was important to rule out that blood contamination was producing our results, although our results in mice indicated an extravascular location. We found a few intravascular HSPCs, confirming previous studies, but the vast majority were extravascular and predominately in vascular-rich zones of the lung alveoli. This anatomic location in the lung is similar to the location of HSPCs in the BM, which are closely positioned next to the vascular sinusoids^33,34^. In the lung, this positioning could be important for seeding of the lung with circulating HSCs and potentially also for the exiting the lung during haematopoietic stress. In this niche, it was notable that lung fibroblasts were in close proximity. The lung mesenchyme is well known to be a critical niche supporting epithelial cell development and repair and similar mechanisms could be operable influencing lung HSPCs^35,36^. Also, we identified a developmental trajectory between lung HSPCs and lung stromal cells. Subpopulations of lung fibroblasts are known to be CD34^+^ and previous lineage-tracing experiments in pulmonary fibrosis have shown a hematopoietic contribution to fibroblasts^37,38^. Future studies are needed in this area to understand how lung HSPCs could be involved in fibrotic lung diseases.

We have not addressed the ontogeny of lung HSPCs—something not possible given the restraints of our human tissue study. There is irrefutable evidence that HSCs commonly enter the circulation and microcirculatory beds, and given the functional and molecular similarities of lung HSPCs to other extramedullary HSPCs, this is perhaps the source of the tissue resident HSPCs in the lung^31,39^. There is precedence, however, for tissue residency to be endowed during development, such as with the yolk-sac derived macrophages^40^. Future studies will be needed to answer this question in mice, including the possibility that hemogenic endothelium in the lung could be the source.

Our findings reframe our understanding of the HSPC pool and its molecular diversity and should enable future studies that could potentially lead to therapeutic advances, such as for life-saving HSC transplantation for BM malignancies and failure. In the modern era, transplantation is mainly accomplished using mobilized HSCs obtained from the peripheral blood. We propose that these mobilized cells are sourced from diverse tissues, including the lung, and that the composition of this pool may be functionally heterogeneous, which could have important implications for treatment responses and complications. In this regard, our findings may also help to understand the mechanisms of leukemogenesis with the possibility that lung HSPCs are direct targets of environmental carcinogens^41^. Further, our findings add to our expanding understanding of rare cell types in the lung and their potential functions^42^, including the possibility of a lineage relationship with lung stromal cells that could be important in fibrosing lung conditions.
